# Home-cage behavior is impacted by stress exposure in rats

**DOI:** 10.3389/fnbeh.2023.1195011

**Published:** 2023-06-09

**Authors:** Evren Eraslan, Magda João Castelhano-Carlos, Liliana Amorim, Carina Soares-Cunha, Ana João Rodrigues, Nuno Sousa

**Affiliations:** ^1^School of Medicine, Life and Health Sciences Research Institute (ICVS), University of Minho, Braga, Portugal; ^2^ICVS/3B's – PT Government Associate Laboratory, Guimarães, Portugal; ^3^Department of Physiology, Faculty of Veterinary Medicine, Istanbul University-Cerrahpasa, Istanbul, Türkiye; ^4^P5 Clinical Digital Center, Braga, Portugal; ^5^Clinical Academic Center (2CA), Braga, Portugal

**Keywords:** social behavior, rats, stress, CUS, enriched environment, home-cage behavior

## Abstract

Being social animals, rats exhibit a range of social behaviors that help them build social bonds and maintain group cohesion. Behavior is influenced by multiple factors, including stress exposure, and the expression of the impact of stress on both social and non-social behaviors may also be affected by the living conditions of rats. In this study, we explored the physiological and behavioral effects of chronic unpredictable stress on group-housed rats in the PhenoWorld (PhW), a socially and physically enriched environment closer to real-life conditions. Two independent experiments were performed: one in the control condition (PhW control, n = 8) and one in the stress condition (PhW stress, n = 8). Control animals remained undisturbed except for cage cleaning and daily handling procedures. Stress group animals were all exposed to chronic unpredictable stress. Data confirm that stress exposure triggers anxiety-like behavior in the PhW. In terms of home-cage behaviors, we found that stress affects social behaviors (by decreased playing and increased huddling behaviors) and non-social behaviors (as shown by the decrease in rearing and walking behaviors). These results are of relevance to expand our knowledge on the influence of stress on social and non-social behaviors, which are of importance to understand better species-typical behaviors.

## 1. Introduction

Providing the social and physical environment to perform necessary species-typical behavior is one of the animal welfare concerns (Jirkof et al., 2019). Laboratory animals are recommended to be housed in social groups (Simpson and Kelly, 2011). Housing conditions, group housing, or housing in pairs in standard cages or enriched environments alter behavioral and neuroendocrine responses to stress (Westenbroek et al., 2005; Smail et al., 2020). Animals exposed to complex, enriched environments, rather than standard laboratory conditions, exhibited neural plasticity and enhanced cognitive performance, positively affected emotional regulation, and showed more adaptive stress responses (Bardi et al., 2016; Lambert et al., 2016). In addition, rats in the enriched environment were reported to exhibit higher levels of sleep behavior and lower levels of agonistic behavior than rats in the unenriched environments (Abou-Ismail et al., 2010), and they demonstrated consistent engagement with conspecifics compared to single or pair housed rats (Pinelli et al., 2017). Moreover, environmental enrichment prevents the development of stereotypes in rodents housed under standard laboratory conditions (Callard et al., 2000; Gross et al., 2012). Previous studies showed that conventional housing of laboratory rodents negatively impacts health parameters in these animals (Lahvis, 2017; Cait et al., 2022). These findings raised concerns about investigating the cognitive and emotional status of animals housed in standard laboratory cages and the suitability of conventional housing conditions for obtaining valid research data (Sherwin, 2004; Lambert et al., 2016).

Rats are social animals and live in groups; interestingly, within a colony, they form social bonds and coordinate group activities (Kramess et al., 1969; Amorim et al., 2022). Social behaviors are those behaviors that involve interactions between two or more individuals, while non-social behaviors are those that are performed by an individual alone. In rats, social behaviors may include activities such as huddling, playing behavior, and communication, while non-social behaviors may include activities such as walking, rearing, self-grooming, eating, drinking, and sleeping (Draper, 1967; Niesink and Van Ree, 1982; Cirulli et al., 1996; Saibaba et al., 1996). They engage in play behavior, such as chasing and rough-and-tumble, which helps them to learn and practice important social skills (Vanderschuren et al., 1997; Schweinfurth et al., 2017; Pellis et al., 2022). Overall, the social behavior of rats is complex and dynamic, and it plays a crucial role in their success as a species. Environmental and social factors have significant effects on social and non-social behaviors (Weyers et al., 1994; Saxena et al., 2021). Our aim in this study was to determine how chronic unpredictable stress (CUS) exposure would affect the social and non-social behaviors of rats housed in an enriched environment and to verify whether these effects of stress are reproduced in animals living in standard housing conditions. Stress is defined as the adaptative and maladaptive responses to an unpredictable/uncontrollable challenge to an individual (Koolhaas et al., 2011). As a stress protocol, we used the CUS protocol which has been widely used to study the impact of stress exposure and consists of random, intermittent, and unpredictable exposure to a variety of stressors lasting at least 4 weeks (Monteiro et al., 2015).

## 2. Material and methods

### 2.1. Animals and experimental design

**Figure 1:**
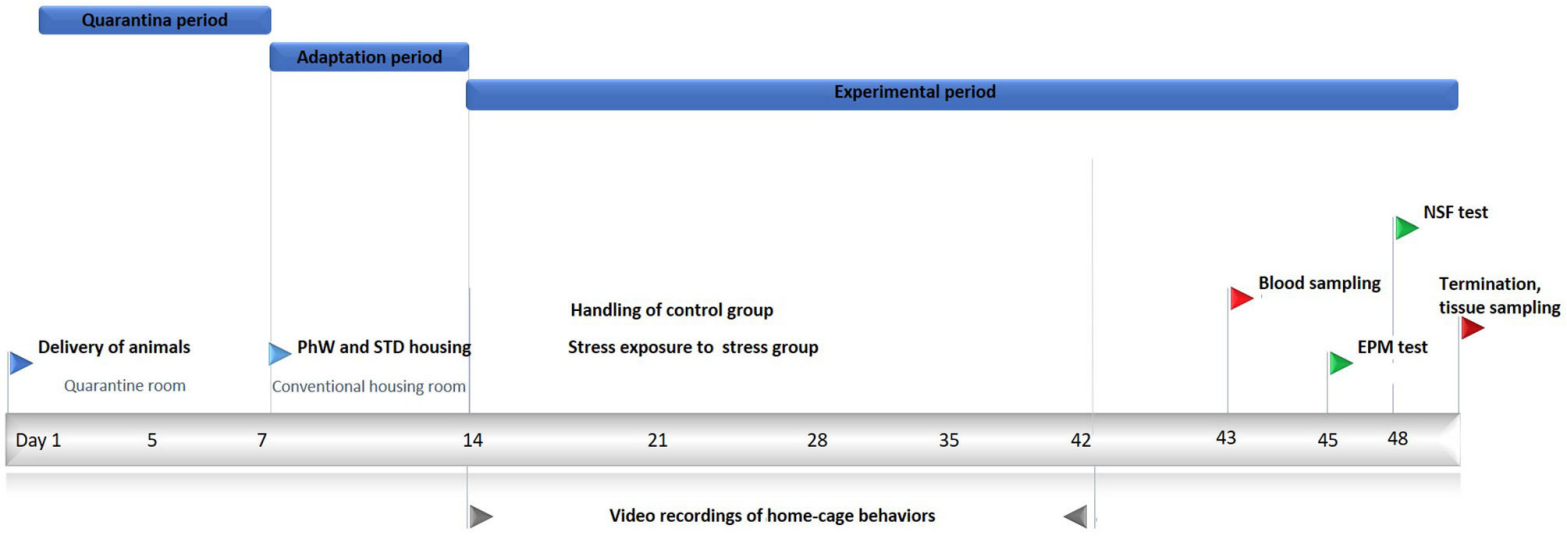
Experimental procedures. Wistar rats aged 7–8 weeks were held in quarantine for 7 days and afterward moved to a conventional housing room. After 7 days of the adaptation period here, the control group was only handled, and the stressed group was exposed to chronic unpredictable stress for 4 weeks. Home-cage behaviors were recorded for a total of 10 days during the experimental period. After this period, blood samples were obtained for corticosterone analyses. EPM and NSF tests were conducted, and the animals were killed at the end of the experiments.

Young adult rats were chosen for the study as social behaviors were known to be more frequently observed in this age group (Klein et al., 2010). Wistar Han male rats, aged 7–8 weeks, were purchased from Charles River Laboratories (Saint Germain Nuelles, France). Animals were kept in a quarantine room for 1 week in groups of four. Then rats were transferred to a standard housing room provided with standard laboratory conditions of an artificial 12/12 light–dark cycle, lights on from 8:00 a.m. to 8:00 p.m., with a relative humidity of 50–60%, and 22°C ambient temperature. They had *ad libitum* access to food and water. They were assigned to two conditions as control and stress groups in eight animals each to be housed in the PhW (TSE Systems GmbH, Bad Homburg, Germany). The PhW setup consists of a 1 m^2^ area and a 50 cm high central cage with corncob bedding on the floor, connected to two drinking/feeding boxes through two open-access tubes. All areas were covered either by perforated Plexiglas or stainless-steel grids. Cardboard tubes were provided to all groups as environmental refinement, and standard type III cages were placed in the central area of the PhW for jumping and climbing (Castelhano-Carlos et al., 2014). Animals had an adaptation period of 1 week in the conventional housing room before initiating the experiments. Two independent experiments were performed: one in the control condition (PhW control, *n* = 8) and one in the stress condition (PhW stress, n = 8). Control animals remained undisturbed except for cage cleaning and daily handling procedures. Stress group animals were all exposed to stress. The same experimenter handled the animals during the study. All experimental procedures are shown in Figure 1. Furthermore, control and stress groups living in standard (STD) cages (animals housed in a group of four in standard cages, which is a standard filter-topped transparent cage 610 × 435 × 215 mm (2,065 cm^2^ floor area) (ref. 2000P, Tecniplast, Buguggiate, Italy) were used to confirm the endocrine and all behavioral effects of stress in distinct housing conditions (Supplementary Figure 1).

All experiments were carried out in accordance with the European Directive 2010/63/EU and the Portuguese regulations and laws (Decreto-Lei 113/2013 and Decreto-Lei 1/2019) on the protection of animals used for scientific purposes of the Ministry for Agriculture, Ocean, Environment and Spatial Planning, which authorized the project in which this study was included (authorization code 9458). The present study was also evaluated and approved by the ethics committee of the University of Minho.

### 2.2. Chronic unpredictable stress

The stress protocol included a chronic unpredictable stress paradigm, which is a validated stress protocol, that was previously described and proven to induce physiological and behavioral alterations typical of the chronic stress response in previous studies and also has been successfully utilized in our laboratory (Sousa et al., 1998; Pêgo et al., 2008; Magalhães et al., 2017; Ventura-Silva et al., 2020). Exposure to this stress protocol is known to induce anxiety-like behavior (Pêgo et al., 2008; Jacinto et al., 2017). One of several stressors was applied in random order and at different intervals of the light phase of the day, daily for 4 sequential weeks (Supplementary Table 1). Stressors were applied in a separate experimental room from where the animals were housed. Unstressed group animals were handled at the same time. The stressors were as follows: cold water (18°C), overcrowding, vibration, restricted space, and exposure to a hot air stream (Table 1). Stressors were applied during the light phase of the day in random order. During stress exposure, PhW animals were randomly separated into groups depending on the type of stressor applied. Weekly body weight and post-mortem adrenal weight were recorded to evaluate the impact of stress exposure.

**Table 1:** Chronic unpredictable stress paradigm.

**Overcrowding**	**Animals were placed in 8 per STD cage (610 × 435 × 215mm) instead of 4 for 1 h**
Restricted space	Four animals living together were confined in STD cages (425 × 266 × 185 mm) for 1 h
Exposure to a hot air stream	Animals were exposed to a hot air stream ranging from 45–50°C for 45 min. PhW animals were placed in STD cages (610 × 435 × 215 mm) in groups of four
Cold water	Replacement of bedding material with cold water, 400 ml (18°C) for 1 h. PhW animals were placed in STD cages (610 × 435 × 215 mm) in groups of four
Vibration	Placement on a vibrating/rocking platform for 15 min

### 2.3. Serum corticosterone

Blood samples were harvested by a tiny incision on a dorsal tail vein at two different time points; within 1 h after lights on and after lights off. Then, the collected blood samples were centrifuged at 13000 rpm for 10 min. The serum was removed and stored at −80°C until analyses.

Serum levels of corticosterone were quantified by the enzyme-linked immunosorbent assays (ELISA) according to the manufacturer's instructions (ADI-900-097, Enzo Life Sciences, Lausen, Switzerland). The absorbance at 405 nm was measured using a microplate reader. The concentration of 26.99 pg/ml corticosterone was the minimum detectable level.

### 2.4. Elevated plus maze

The anxiolytic-like behaviors of animals were assessed in an elevated plus maze (EPM). The EPM apparatus (ENV-560; Med Associates Inc., St. Albans, VT, USA) was a black polyproline plus-shaped platform with two open (50.8 × 10.2 cm) and two closed (50.8 × 10.2 × 40.6 cm) arms, heightened 72.4 cm above the floor. The junction area between the four arms measured 10 × 10 cm. A raised edge (0.5 cm) on the open arms provided additional grip for the rats. The experimental room was lit by 40 W fluorescent lamps mounted above the maze so that all arms were equally illuminated (300 lx at the maze floor level). A charge-coupled device (CCD) camera placed above the maze recorded the behaviors. Animals were placed in the center of the apparatus facing one of the open arms and tested for 5 min. The maze was cleaned using ethanol solution (70%) and wiped dry between trials to eliminate any odor cues. Time spent in closed and open arms, the central area, and the number of entries into each arm of the maze were obtained by behavioral observation of recorded video tapes.

The percentages of time spent in the open arms (100 × time spent in the open arms/total time spent in the open and closed arms), and also the percentage frequency of entries in the open arms (100 × number of entries into open arms/total entries into all arms), and total arm entries (total number of closed and open arm entries) were calculated as an index of anxiety-like behavior.

### 2.5. Novelty-suppressed feeding

The novelty-suppressed feeding test (NSF) was also used to measure anxiety-related behaviors as previously described (Bodnoff et al., 1988; Alves et al., 2017). Animals were food-deprived for 24 h. The rats were transferred to the testing room 2 h before the NSF test, and the testing was done under housing illumination conditions. Animals were subsequently individually placed in the corner of a square open field arena containing a single food pellet in the center on a circular white paper. An amount of sawdust covered the floor and was mixed after each trial to eliminate olfactory stimuli. The duration of the test was 5 min. The latency to feed, which was defined as chewing the food, not simply sniffing or playing with a pellet, was measured and used as an index of anxiolytic behavior. Shorter latency to eat the pellet in a novel environment was interpreted as lower anxiety-like behavior. After reaching the pellet or when animals had not eaten within 300 s, the test was terminated.

### 2.6. Observation of home-cage behaviors

The behaviors of rats were recorded in their home cages by surveillance video cameras installed above PhW. The time-sampling model, measuring behavior over a limited time at present intervals, was used for the scoring (Saibaba et al., 1996; McCormick et al., 2007). An arbitrary time window of the 1-h period was chosen for all groups in each observation day within the first 2 h of the dark phase. Behaviors of each animal were scored as frequencies within the first 5 min of every 10-min interval for 1 h (5 × 6 = 30 min) performed for 10 days (300 min) for a total of 10 different observation periods chosen from video recordings during the experimental period (days 14–42). The results were monitored across a total of 10 days, 2–3 days observation period in each week, and pooled into a single value (mean) (the effect of time between the observation days was not significant) per rat for each behavior pattern.

The observed behaviors were classified into two categories: social activities including social play, following, social investigation, and huddling, and non-social activities such as self-grooming, walking, rearing, and digging (Draper, 1967; Niesink and Van Ree, 1982; Cirulli et al., 1996; Saibaba et al., 1996; Vanderschuren et al., 1997) (Supplementary Table 2).

All videos of behaviors were scored by the same observer, and the second observer scored part of the videos to control for a possible bias.

### 2.7. Post-mortem verifications

At the end of the study, all rats were killed by decapitation under intraperitoneal sodium pentobarbital (20% Eutasil^®^, Sanofi, Gentilly, France) anesthesia. A necropsy was performed, and the adrenal glands were taken out, cleaned out of the surrounding tissues, and weighed (PR503, Mettler Toledo). The weight of the adrenal glands was then divided by the weight of the rat to assess “relative weight,” and these data were used for later analyses.

### 2.8. Statistics

Data were initially evaluated for normality using the Shapiro–Wilk test. Parametric or non-parametric tests were used depending on the normality of the data. Statistical comparisons were conducted to compare the control group vs. the stress group. Body weight gain was analyzed by repeated-measures analyses of variances (ANOVA) regarding stress as between-subject factors and days as within-subject factors. Two different time points (nadir vs. zenith) of corticosterone levels were analyzed by repeated-measures ANOVA. An independent sample *t-*test was conducted to compare the main effects of stress on adrenal weight, each of the sampling points of corticosterone measurements, EPM, NSF, home-cage behaviors, and the non-parametric Mann–Whitney U-test was performed when the data were not normally distributed.

The level of statistical significance was set to a *p*-value of ≤ 0.05. The data presented in the graphs indicate group mean ± SEM. All analyses were performed using SPSS software version 21 (SPSS Inc., Chicago, IL, USA).

## 3. Results

### 3.1. Body weight gain (%)

**Figure 2:**
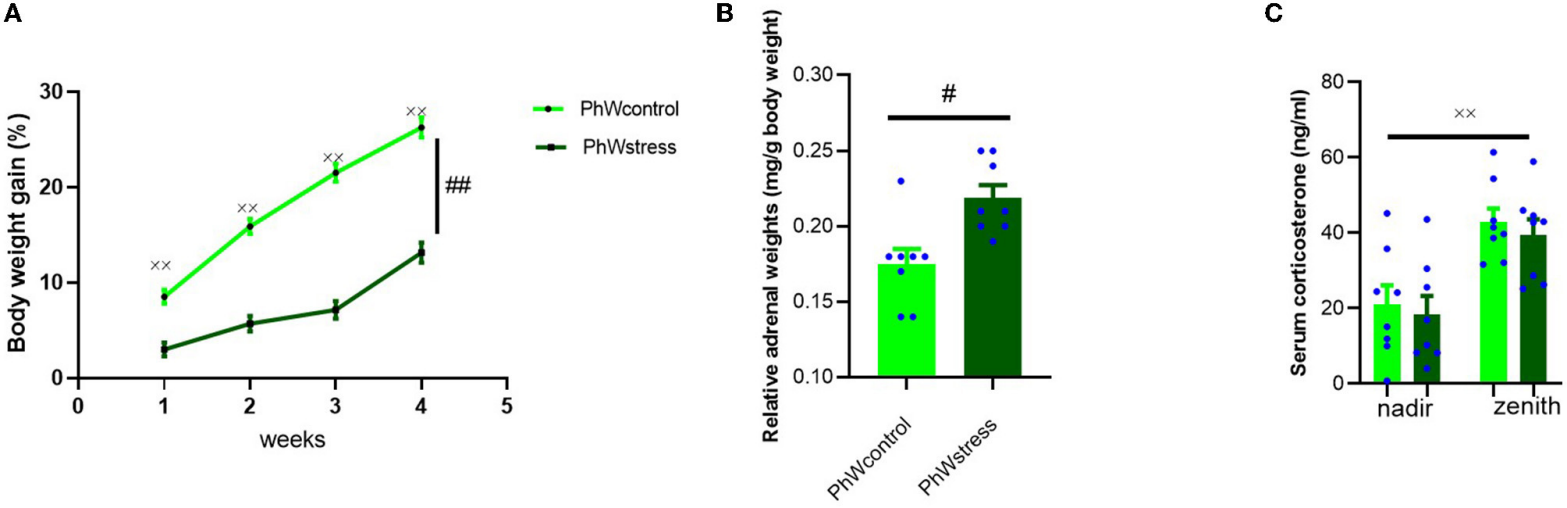
**(A)** Body weight gain (%) of animals during the experiment. A significant effect of time and stress was observed. **(B)** Relative adrenal weight of animals (mg/g body weight). **(C)** Serum corticosterone levels (ng/ml) at the end of the experimental period at nadir (8–9 a.m.) and zenith (20–21 p.m.). Data are presented as mean ± s.e.m; ^xx^*p* < 0.001, ^#^*p* < 0.05, and ^##^*p* < 0.001 indicating the general effects of time and stress, respectively.

All animals gained weight during the experimental period (*F* (3.42) = 223.31, *p* < 0.001). Control animals gained more weight than stressed animals (*F* (1.15) = 103.27, *p* < 0.001) (Figure 2A).

STD cage animals' body weight gain also increased over time (*F* (3.54) = 122.31, *p* < 0.001), and stressed animals gained less weight than controls during the experiment (*F* (1.18) = 8.38, *p* = 0.010) (Supplementary Figure 1A).

### 3.2. Hormonal measurements

#### 3.2.1. Adrenal weights

Adrenal weights were corrected for body weight and were used as a surrogate marker of response to CUS. There was a significant effect of stress exposure on relative adrenal weights. Animals exposed to stress had higher adrenal weights (*t* (14) = −3.33, *p* = 0.005) (Figure 2B).

Relative adrenal weights of stressed animals in STD cages were also higher than those of controls (*t* (18) = −4.10, *p* = 0.001) (Supplementary Figure 1B).

#### 3.2.2. Corticosterone levels

To obtain further insight into the impact of stress exposure in this study, we determined the corticosterone levels at the nadir and zenith. As expected, corticosterone levels of animals were lower when the lights were on (8–9 a.m.) and higher with the lights off (8–9 p.m.) (Allen-Rowlands et al., 1980; Castelhano-Carlos et al., 2014) in all conditions (*F* (1.15) = 50.17, *p* < 0.001). No significant differences were found between stress-exposed rats and controls (Figure 2C).

The results were similar for STD animals; corticosterone levels of animals were lower at the nadir than at the zenith (*F* (1.14) = 11.75, *p* = 0.004). The difference between stressed and control animals was insignificant (Supplementary Figure 1C).

#### 3.2.3. Anxiety-like behavior

**Figure 3:**
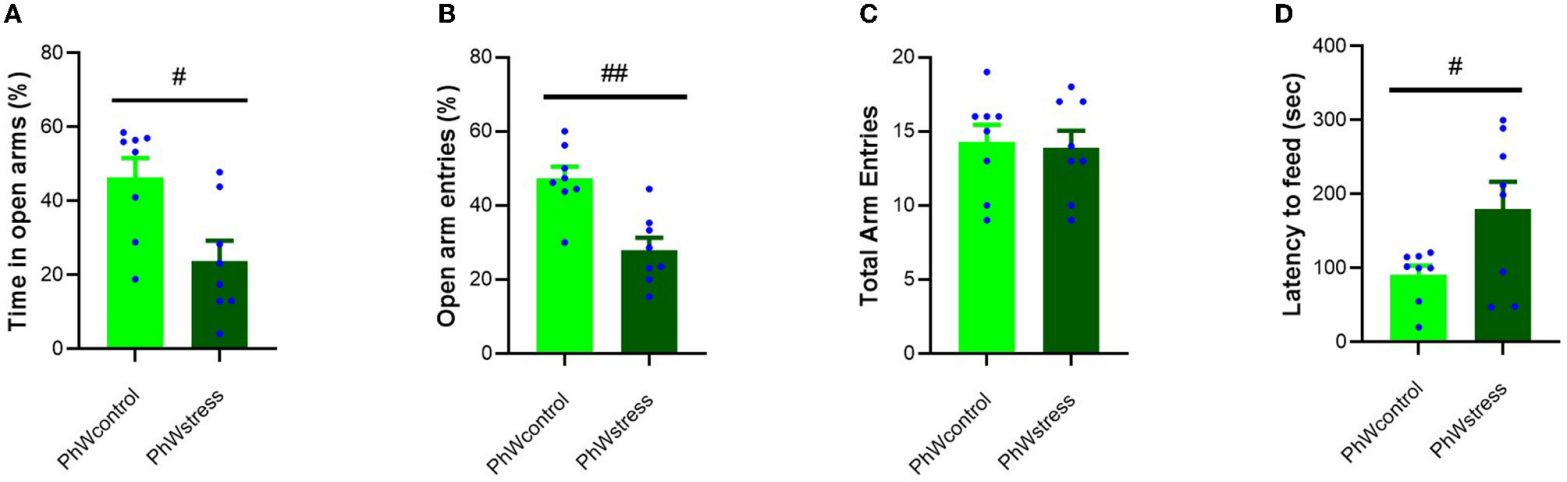
Behavioral data obtained in the EPM and NSF. **(A)** Percentage of time spent in the open arms. **(B)** Percentage of open arm entries. **(C)** Total arm entries of animals. **(D)** Latency to feed (sec) in NSF test. Data are presented as mean ± s.e.m; ^#^*p* < 0.05 and ^##^*p* < 0.001 indicating the effect of stress.

Stress exposure significantly affected animals' anxiety-like behavior when considering the percentage of time spent in open arms of the elevated plus maze (EPM). Stressed animals spent less time in open arms than control animals (*t* (14) = 2.94, *p* = 0.011; Figure 3A), and the ratio of open/total arm entries of stressed animals was lower than that of control animals (*t* (14) = 4.18, *p* = 0.001; Figure 3B). Total arm entries of animals were not affected by stress treatment (Figure 3C). In the novelty-suppressed feeding test (NSF), the latency to feed time was higher in stressed animals compared to controls (*t* (14) = −2.30, *p* = 0.037) (Figure 3D).

Of note, these alterations found in anxiety-like behavior, both in the EPM and in the NSF, were not reproduced in animals living in STD cages (Supplementary Figures 1D, E).

### 3.3. Home-cage behaviors

#### 3.3.1. Social activities

**Figure 4:**
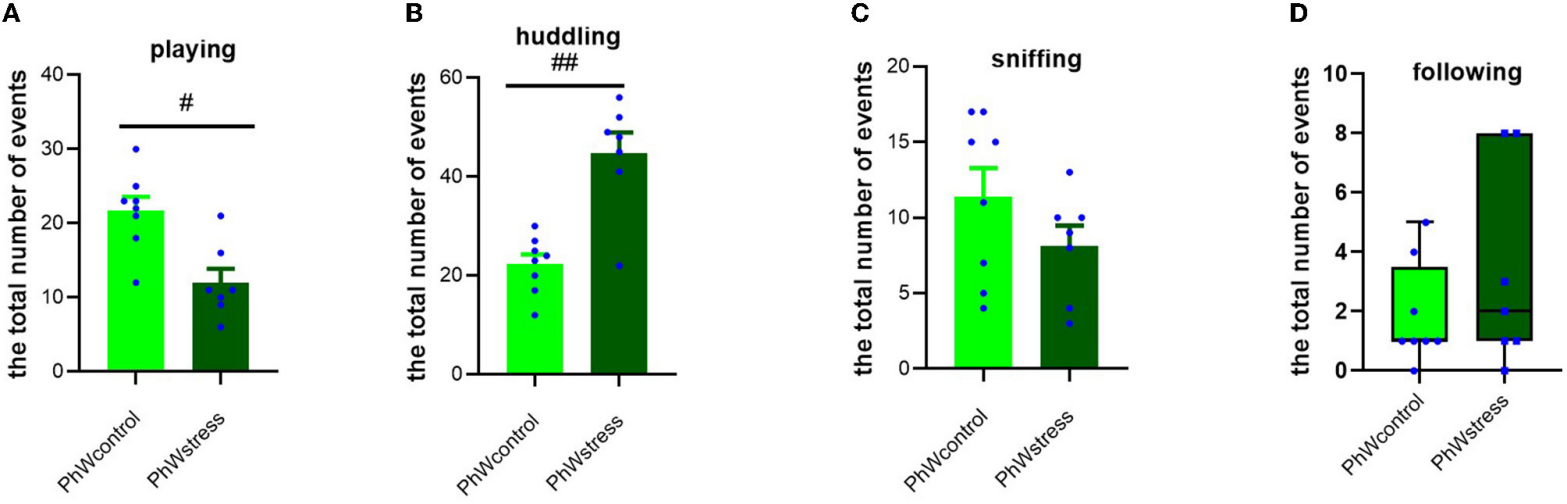
Analyses of social activities in home-cage behaviors (the total number of events). **(A)** Social-play behavior. **(B)** Huddling behavior. **(C)** Sniffing behavior. **(D)** Following behavior. Normally distributed data are presented as mean ± s.e.m; ^#^*p* < 0.05 and ^##^*p* < 0.001 indicating the effect of stress exposure. Non-normally distributed data are presented by box plots where the central lines represent the median, and the whiskers represent the minimum and maximum values.

Stress exposure had a significant effect on playing and huddling behaviors. Control animals had higher scores of playing (*t* (13) = 3.69, *p* = 0.003; Figure 4A) and lower scores of huddling (*t* (13) = −5.02, *p* < 0.001; Figure 4B) than stress group animals. Sniffing and following behaviors were not impacted by CUS (Figures 4C, D).

In animals housed in STD conditions, we confirmed the impact of stress on social behavior, both in the playing (*Z* = −2.42, *p* = 0.015) and huddling behavior (*t* (14) = −3.28, *p* = 0.005) and also in sniffing behavior (*Z* = −2.17, *p* = 0.028), where stressed animals had lower scores than controls (Supplementary Figure 1F).

#### 3.3.2. Non-social activities

**Figure 5:**
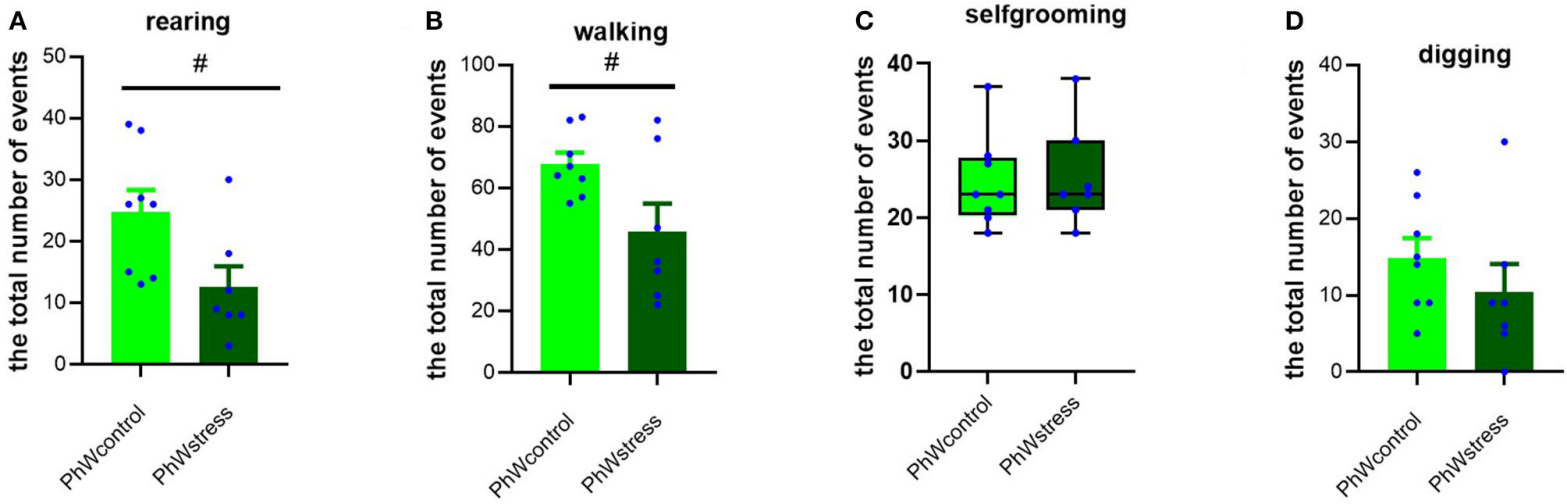
Non-social behaviors in the home cage. **(A)** Rearing behavior. **(B)** Walking behavior. **(C)** Self-grooming behavior. **(D)** Digging behavior. Normally distributed data are presented as mean ± s.e.m; ^#^*p* < 0.05 indicating the effect of stress exposure. Non-normally distributed data are presented by box plots where the central lines represent the median, and the whiskers represent the minimum and maximum values.

Considering non-social activities, we found that stress treatment had a significant effect on rearing and walking behaviors. Control animals had higher scores of rearing (*t* (13) = 2.43, *p* = 0.030) and walking (*t* (13) = 2.34, *p* = 0.036) than stressed animals (Figures 5A, B). Self-grooming and digging behaviors did not differ between the control and stress groups (Figures 5C, D).

Importantly, in animals housed in STD conditions, we could not confirm the impact of stress on non-social behavior, as the decreases in rearing and walking in stressed rats were not statistically significant. Only self-grooming behavior was higher in control animals (*t* (14) = −2.34, *p* = 0.034) (Supplementary Figure 1G).

## 4. Discussion

Stress exposure triggers several effects on the body. Herein, in line with previous literature, we show that exposure to CUS led to a reduction in body weight gain and an increased relative adrenal weight, reflecting the impact of the procedure (Sousa et al., 1998; Cullinan and Wolfe, 2000; Westenbroek et al., 2005). On the other hand, the corticosterone levels of animals were not different between groups at the end of the experiment. We may fail to capture the dynamic nature of the HPA axis drive by only conducting end-point hormone sampling. As a result of repeated exposure to stressors, predictability and controllability might have been increased. Therefore, we can interpret normal levels of corticosterone at the end of the experiment as an adaptation rather than a pathology (Koolhaas et al., 2011). However, chronic changes in HPA axis drive can also be assessed *via* increased adrenal weight (Ulrich-Lai et al., 2006). Thus, these physiological changes in body weight gain and adrenal weight are consistent with stress exposure (Smail et al., 2020). The absence of neuroendocrine alteration in response to CUS could not be interpreted as a failure to respond to CUS as stress exposure did impact the body weight gain, adrenal weight, and anxiety-like behavior (Monteiro et al., 2015). Anxiogenic effects of CUS were confirmed both in the EPM and the NSF tests; however, noticeably, these effects were only found in animals living in the PhW indicating that enriched environments, in the case of the PhW, increase the sensitivity of detecting changes in the results of experiments compared to standard housing conditions (Castelhano-Carlos et al., 2014). Research on the effects of enriched environment procedures on anxiety-like behavior of rodents is inconsistent possibly due to variability in enrichment conditions, duration of enrichment, species, strain, sex, and age of animal subjects (Hendershott et al., 2016). Increased anxiety-like behaviors after enrichment were explained by the animals' better adaptability to their test environments and adaptive survival strategies when confronted with a novel, potentially threatening environments (Connors et al., 2015; Kentner et al., 2018). However, reduced anxiety, or no effects of enrichment on anxiety-like behaviors, in EPM were also reported (Simpson and Kelly, 2011; Pinelli et al., 2017).

The main objective of the present study was, however, to compare the effects of stress exposure on home-cage behaviors. We opted to distinguish the home-cage behavior into two categories: social- and non-social behaviors. We aimed to test the stress impact on these behaviors of animals living in an enriched environment, such as the PhW. We found that stress exposure affected both social and non-social behaviors in the PhW. Regarding the social activities of rats, our data reveal that CUS affected playing behavior and huddling. While stress inhibited playing, the huddling time was increased; these changes were reproduced also in animals living in STD conditions. These changes in playing behavior triggered by stress treatment are in line with previous studies (Klein et al., 2010; Muroy et al., 2016; Saxena et al., 2021). Stress-induced inhibition in play behavior is a foreseen outcome of stress exposure, as playing behavior is usually observed under positive environmental conditions and is suppressed by a negative effect (Ahloy-Dallaire et al., 2018; Jirkof et al., 2019). However, it is important to note that different results have also been reported; one study showed that while acute stressors led to a complete, yet transitory, inhibition in social play behaviors, repeated exposure to that stressor did not have any effect (Klein et al., 2010). Similarly, play behavior was completely inhibited following acute stressors in prepubertal male rats but not in adults (Romeo et al., 2006).

Interestingly, we observed an increase in huddling behavior, and this might be associated with the social-coping mechanism of the stressed animals, as was previously reported (Klein et al., 2010; Han et al., 2020). The stressed rats' preference for passive social interactions, i.e., huddling rather than actively playing, might be associated with seeking social support to cope with and recover from the adverse effects of stress (Muroy et al., 2016; Han et al., 2020). Defensive aggregation (e.g., huddling is a response in prey species to a predatory threat) is considered to have a survival benefit (Bowen et al., 2013). Furthermore, huddling is defined as a form of pro-social behavior and, thus, the increase in huddling behavior observed after stress exposure can be interpreted as a potential sign of greater affiliation and social bonding (Muroy et al., 2016; Crockford et al., 2017) and a strategy to cope with the negative effects of stress exposure. In contrast, sniffing and following behaviors were not significantly impacted by stress exposure in animals living in the PhW.

Considering non-social behaviors, we observed that rearing and walking behaviors were less observed in stressed animals in PhW. Curiously, these findings were not observed in animals living in standard housing, which emphasizes the relevance of the environmental factors for the expression of specific behaviors. The analysis of rearing and walking behaviors provides valuable information about several components, including motor function and activity, as well as exploratory behavior. The latter, herein confirmed to be impacted by stress treatment, is of interest as it provides a clear indication of how stress affects adaptation to the environment.

Previously, it has been reported that stress has a divergent effect on social and non-social behaviors, while restraint stress exposure induced suppression in play behavior and increased huddling behaviors and did not change non-social behaviors (Klein et al., 2010). However, the results of studies are controversial in this matter. It was reported that acute immobilization stress increased huddling behaviors but suppressed exploratory activities (Muroy et al., 2016). Similarly, while 2 h of immobilization stress impaired social behaviors (sniffing, nose-to-nose contact) earlier in time, an inhibition in non-social activities (exploratory rearing) was observed 10 days later (Saxena et al., 2021). Therefore, the role of stress exposure in affecting social and non-social behaviors is sensitive to various factors such as duration, the intensity of the stressor, housing conditions of animals, and timing of evaluation of behaviors. In our study, the effect of housing conditions was much stronger in non-social home cage behaviors.

Of note, given the observation that the housing conditions have impacted the results, it should be considered that the provision of a more naturalistic environment, at least enough space to live, and group housing is relevant in the experimental design of behavioral research. For this purpose, various automated and enriched environments have been described to evaluate rodents' behavior to enable long-term evaluations without human interference to improve the validity and reliability of the research results.

## 5. Conclusion

In the study, we assessed the impact of stress on home-cage behavior, which provides insights into rats' natural social and non-social behavior. We demonstrated that both stress exposure and housing conditions impact home cage behaviors, distinctly, while stress treatment affects both social and non-social components of home cage behaviors; housing conditions significantly influence non-social home cage behaviors. These findings contribute to a better understanding of the factors that may influence rats' behavior and report on the best housing and enrichment strategies to promote rats' wellbeing, which can improve the quality of research results.

## Data availability statement

The raw data supporting the conclusions of this article will be made available by the authors, without undue reservation.

## Ethics statement

The animal study was reviewed and approved by the Ethics Committee of the University of Minho.

## Author contributions

NS, AR, and EE contributed to the conception and design of the study. NS provided the funding. EE, MC-C, LA, CS-C, and AR performed experimental analyses and data collection. NS and EE wrote the sections of the manuscript. All authors contributed to the experimental design, manuscript revision, and the final manuscript.
