# Supplementary material for: Home-cage behavior is impacted by stress exposure in rats
Source: Front Behav Neurosci. 2023 Jun 9;17:1195011. doi: 10.3389/fnbeh.2023.1195011 (PMC10288110; doi:10.3389/fnbeh.2023.1195011)
Supplement: Supplementary file 1 [file Data_Sheet_1.docx]

Supplementary Material

Home-cage behavior is impacted by stress exposure in rats

Evren Eraslan*, Magda João Castelhano-Carlos, Liliana Amorim, Carina Soares-Cunha, Ana João Rodrigues, Nuno Sousa

*** Correspondence:** Nuno Sousa: njcsousa@med.uminho.pt

# Supplementary Figures and Tables

## Supplementary Tables

**Table 1** Application schedule of chronic unpredictable stress

| **Dates** | **Time** | **Stress** | **Date** | **Time** | **Stress** |
| --- | --- | --- | --- | --- | --- |
| Day 1 | 15:10- 16:10 | Restricted space | Day 15 | 14:15- 15:15 | Overcrowding |
| Day 2 | 13:15- 13:30 | Vibration | Day 16 | 10:00- 10:45 | Hot air stream |
| Day 3 | 17:30- 18:30 | Overcrowding | Day 17 | 15:30- 16:30 | Cold water |
| Day 4 | 12:30-13:30 | Cold water | Day 18 | 12:25- 13:25 | Restricted space |
| Day 5 | 9:45- 10:30 | Hot air stream | Day 19 | 14:15- 15:00 | Vibration |
| Day 6 | 16:30- 17:30 | Overcrowding | Day 20 | 11:15- 12:15 | Overcrowding |
| Day 7 | 11:40- 12:20 | Vibration | Day 21 | 13:00- 13:45 | Hot air stream |
| Day 8 | 9:50- 10:50 | Cold water | Day 22 | 16:30- 17:30 | Cold water |
| Day 9 | 17:25- 18:10 | Hot air stream | Day 23 | 12:00- 13:00 | Restraint |
| Day 10 | 15:30- 16:30 | Restricted space | Day 24 | 14:15- 14:55 | Shaking |
| Day 11 | 14:30- 15:30 | Overcrowding | Day 25 | 11:15- 12:10 | Cold water |
| Day 12 | 12:05- 12:55 | Vibration | Day 26 | 17:00- 18:00 | Overcrowding |
| Day 13 | 17:00- 18:00 | Restricted space | Day 27 | 9:30- 10:15 | Hot air stream |
| Day 14 | 11:00- 12:00 | Cold water | Day 28 | 15:00- 16:00 | Restricted space |

**Table 2** Ethogram’s of rat behavior in the home-cage

| **Behavioral Element** | **Description** |
| --- | --- |
| Social play | One animal approach and soliciting another (pouncing; attempt to nose or rub of the neck of the partner), chasing the partner, crawling over/under, boxing, wrestling, pinning (lying with the dorsal body surface on the floor with the other animal standing over it), and lateral display |
| Following | Walking or running in the direction of the partner moving away |
| Social investigation (sniffing) | Sniffing of the any part of the body of the cage-partner |
| Huddling | The presence of at least 2 animals, resting or sleeping while maintaining close physical contact with the partner |
| Self-grooming | Repeatedly cleaning the body fur using forelimbs beginning with the snout, progressing to the ears and ending with whole body grooming |
| Walking | Leg movement enabling the animal's slow pace across the floor |
| Rearing | Standing on the hind paws and stretching the body while sniffing the air from the upper part of the cage |
| Digging | Pushing, pulling or kicking bedding material away and around using the snout and/or both the forepaws and hind paws |

## Supplementary Figures

**
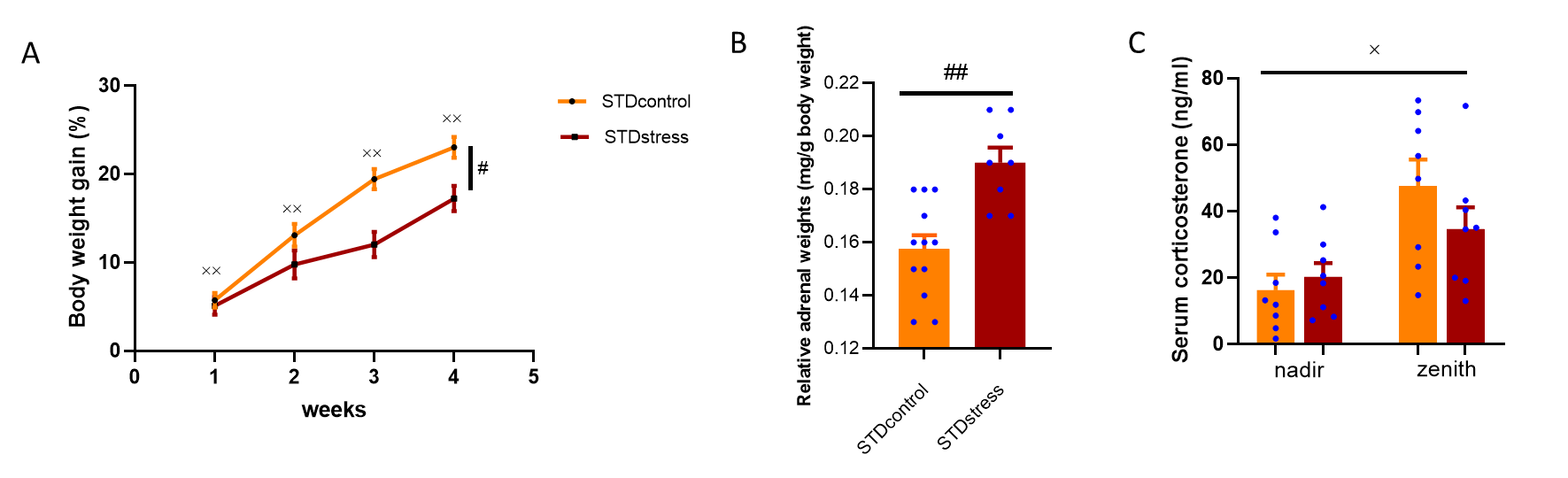
**

**
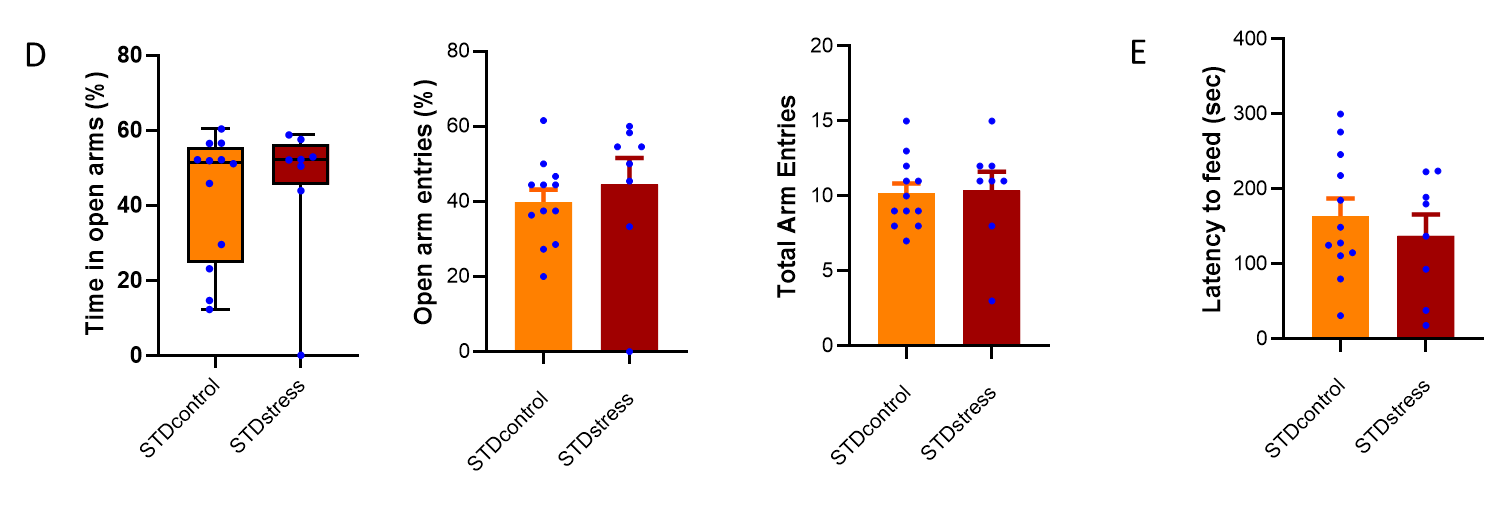
**

**
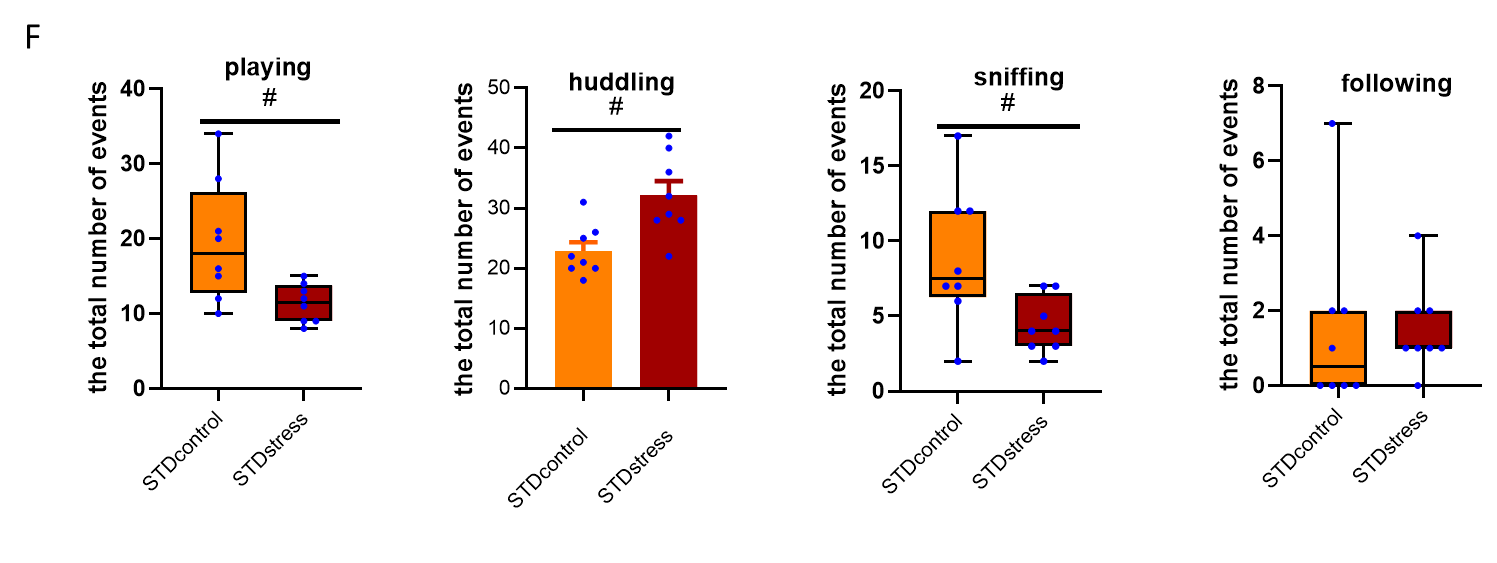
**

**
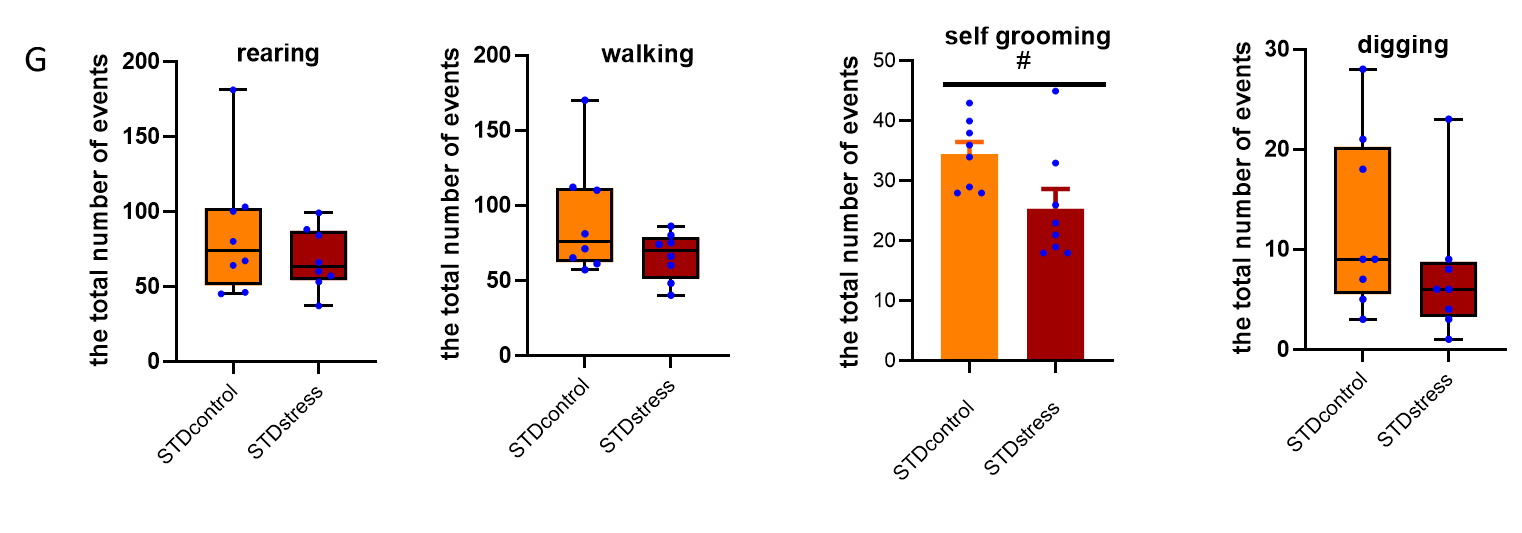
**

**Supplementary Figure 1.** Data obtained from animals living in STD cages **A)** Body weight gain (%) of STD cage animals during the experiment **B)** The relative adrenal weight of animals (mg/g body weight) **C)** Serum corticosterone levels (ng/ml) at the end of the experimental period at nadir (8-9 a.m.) and zenith (20-21 p.m.) **D)** Behavioral data obtained in the elevated plus maze (EPM) **E)** Anxiety-like behaviors in the novelty suppressed feeding test (NSF) **F)** Analyses of social activities in home-cage behaviors (the total number of events for social play, huddling, sniffing and following behaviors) **G)** Non-social activities in home-cage behaviors (the total number of events for rearing, walking, self-grooming and digging behaviors). Normally distributed data is presented as means ± s.e.m. Non-normally distributed data is presented by box plots; the central lines represent the median, the whiskers represent the minimum and maximum values. ^x^*p* < 0.05, ^xx^*p* < 0.001 and ^#^*p* < 0.05, ^##^*p* <0.001 indicate the general effect of time and stress, respectively.
